# Alpha-estradiol and (R)-(−)-ibuprofen inhibit gastric cancer progression via GLI1 G-quadruplex

**DOI:** 10.3389/fphar.2025.1492694

**Published:** 2025-04-04

**Authors:** Qiang Li, Pan Pan, Qingqing Xian, Jingtan Li, Jingting Wang, Jiaying Cai, Jing Wang, Yanfei Jia, Haiji Sun, Lulu Zhang, Xiaoli Ma

**Affiliations:** ^1^ Research Center of Basic Medicine, Central Hospital Affiliated to Shandong First Medical University, Jinan, Shandong, China; ^2^ Department of Pathology, Central Hospital Affiliated to Shandong First Medical University, Jinan, Shandong, China; ^3^ College of Life Science, Shandong Normal University, Jinan, Shandong, China

**Keywords:** G-quadruplex, GLI1, alpha-estradiol, (R)-(−)-ibuprofen, PRKACB, gastric cancer

## Abstract

**Background:**

The transcription factor GLI1, aberrantly activated in gastric cancer, drives tumor progression, yet no approved inhibitors currently target this molecule. G-quadruplex (G4) motifs in promoter regions have emerged as promising therapeutic targets. This study explores G4 stabilization in the GLI1 promoter as a novel strategy to suppress gastric cancer progression.

**Methods:**

G4 formation in the GLI1 promoter was validated using circular dichroism. A dual-luciferase assay screened FDA-approved drugs for G4-stabilizing activity, identifying alpha-estradiol and (R)-(-)-ibuprofen as candidates. These compounds were evaluated for anti-tumor effects through in vitro assays (proliferation, migration, invasion) and in vivo xenograft models. Mechanistic insights into GLI1/PRKACB signaling were obtained via chromatin immunoprecipitation and pathway analysis.

**Results:**

Stable G4 structures were confirmed in the GLI1 promoter. Alpha-estradiol and (R)-(-)-ibuprofen suppressed GLI1 transcription and protein levels, significantly inhibiting gastric cancer cell proliferation, migration, invasion, and stemness. In vivo, both compounds reduced tumor growth and metastasis, with (R)-(-)-ibuprofen synergizing with cisplatin to enhance efficacy. Mechanistically, GLI1 directly regulated PRKACB expression, and G4 stabilization downregulated PRKACB, impairing epithelial-mesenchymal transition and cancer stemness.

**Conclusion:**

Targeting GLI1 G4 structures with alpha-estradiol and (R)-(-)-ibuprofen effectively inhibits gastric cancer progression by blocking GLI1/PRKACB signaling. This study highlights G4-targeted therapy as a novel and clinically translatable strategy for gastric cancer treatment.

## 1 Introduction

Gastric cancer is the fifth most commonly diagnosed cancer and the fourth leading cause of cancer-related death in the world (Sung et al., 2021). Due to dramatic advancements in *Helicobacter pylori* eradication, nutritional improvement, earlier diagnosis, and therapeutic breakthroughs, the incidence of gastric cancer has declined over the past few decades (Park and Herrero, 2021). However, there is an unmet clinical demand for targeted therapies for advanced gastric cancer (Smyth et al., 2020). Therefore, the identification of novel therapeutic targets and elucidation of the underlying mechanisms are urgently needed in gastric cancer.

Glioma-associated oncogene homolog 1 (GLI1) is an important transcription factor downstream of the Hedgehog (Hh) signaling pathway and can be used as a marker of Hh signaling pathway activation (Wang et al., 2019; Jagani et al., 2010). The Hh/GLI signaling pathway is aberrantly activated in various tumors, including gastric cancer (Qi et al., 2020), lung adenocarcinoma (Li et al., 2016), oral squamous carcinoma (Shimo et al., 2016), breast carcinoma (Hegde et al., 2008), and hepatocellular carcinoma (Yan et al., 2016), and is closely associated with tumorigenesis, progression, invasion, and metastasis. In addition, studies (Aberger et al., 2012; Kern et al., 2015) have found that the Hh/GLI signaling pathway can promote tumor progression through other signaling pathways through a cross-regulatory phenomenon. Various small-molecule inhibitors of the Hh pathway are currently being developed; however, there are two major problems. Most inhibitors have been developed to target PTCH1 and SMO (Xu et al., 2019). Previous antitumor studies have focused on SMO targets upstream of the Hh pathway; however, in recent years, GLI has been found to play a more important role than SMO in cancer development (Stecca and Ruiz i Altaba, 2010). Second, only two drugs (GDC-0449 and LDE225) have been approved by the FDA for the treatment of basal cell carcinoma and medulloblastoma, and no molecular inhibitors have been approved for the treatment of gastric cancer (Rubin and de Sauvage, 2006; Bariwal et al., 2019). Therefore, blocking or silencing the expression of GLI1 will significantly help suppress gastric cancer progression.

G-quadruplexes are a class of guanine-rich DNA or RNA secondary structures stabilized by Hoogsteen hydrogen bonds (Nishio et al., 2021). Many potential G-quadruplex formation motifs are present in the human genome. G-quadruplex structures regulate genomic stability, DNA replication, and gene expression, many of which are involved in oncogenesis (Awadasseid et al., 2021). Therefore, searching for ligands that can induce G-quadruplex formation in guanine-rich regions of DNA and screening for potential compounds as anticancer drugs represent research hotspots in the field of cancer therapy (Tassinari et al., 2021). Through *in vitro* experiments, we verified sequences that could form a G-quadruplex structure and thus could be used as molecular targets for inhibitor development.

cAMP-dependent protein kinase catalytic subunit β (PRKACB) mediates the cAMP-dependent pathway associated with various cellular processes such as cell proliferation, cell cycle, chromatin condensation, nuclear membrane reorganization, and intracellular transport mechanisms (Wang et al., 2020). Recently, it was found that PRKACB is likely associated with the epithelial mesenchymal transition (Falch et al., 2018). PRKACB has two isoforms, Cβ1 and Cβ2, of which the Cβ1 isoform is abnormally expressed in gastric cancer tissues (Furuta et al., 2012). PRKACB regulates p75, PKA, and other key molecules involved in cell survival, differentiation, metabolism and apoptosis (Kamilaris et al., 2020). Excessive activity can cause diseases such as adrenal tumors (Espiard et al., 2018). Therefore, PRKACB has an important physiological function; however, in gastric cancer, the function of PRKACB is not clear, and its upstream and downstream regulatory molecular pathways are rarely reported.

Here, we studied the effects of G-quadruplex regulators of the GLI1 promoter on gastric cancer progression. Furthermore, this effect was confirmed by inhibition of the GLI/PRKACB pathway in gastric cancer.

## 2 Materials and methods

### 2.1 Online database analysis

The expression of GLI1 and its correlation with clinical stage and lymph node metastasis of gastric cancer were analyzed using the online databases GEPIA (http://gepia.cancer-pku.cn) and UALCAN (http://ualcan.path.uab.edu/index.html), respectively. The correlation between GLI1 and PRKACB and their clinical significance with overall survival (OS) was analyzed using TIMER (https://www.timeanddate.com/timer) and The Human Protein Atlas (https://www.proteinatlas.org/).

### 2.2 Tissue specimens and cell culture

The tissue microarray (HStm-Muc060CS-01) consists of 30 adenocarcinoma and 30 tumor-adjacent tissue samples. Each group of paired carcinoma and tumor-adjacent tissues was collected and classified according to the clinical information. Twenty of the 30 specimens were from males and 10 were from females, ranging from to 45–82 years old (mean age, 61 years). The tissues of the patient specimens were obtained from the Department of Pathology, Central Hospital, affiliated with Shandong First Medical University.

The human gastric epithelial cell line GES-1, gastric adenocarcinoma cell line HGC-27, and AGS were purchased from IBCB (Shanghai Institute of Biochemistry and Cell Biology, Chinese Academy of Science). Cells were grown in RPMI 1640 medium (HyClone) and Ham’s F-12 nutrient medium supplemented with 10% fetal bovine serum (FBS) (Gibco) and 1% penicillin and streptomycin (Macgene). Cells were cultured at 37°C in a humidified atmosphere containing 5% CO_2_. Human gastric cancer cell lines were treated with 20 µM GANT61 (an inhibitor of GLI1), 1 µM alpha-estradiol or (R)-(−)-Ibuprofen for 24 h.

### 2.3 Cell transfection

AGS and HGC-27 cells were seeded in 6-well plates at a density of 5 × 10^5^ cells per well and cultured overnight to reach 70%–80% confluence. Then the cells were transfected with the PRKACB plasmid in opti-MED medium using Lipofectamine 2000 Transfection Reagent at room temperature, according to the manufacturer’s protocol. 24 h after transfection, PRKACB expression in AGS and HGC-27 cells was assessed by Western blot analysis.

### 2.4 Immunohistochemistry

Immunohistochemical staining was performed using a streptavidin-peroxidase assay. Tissue sections were cut from the formalin-fixed paraffin-embedded tissue blocks at a thickness of 4 µm and mounted on glass slides.The slides were then dewaxed in xylene for 10 min twice and rehydrated through a series of graded ethanol solutions (100%, 95%, 80%, 70%) for 5 min each. Antigen retrieval was carried out by boiling the slides in a citrate buffer (pH 6.0) in a microwave oven for 10 min. Then, the slides were incubated with primary antibodies: anti-GLI1 MAb (1:200; Proteintech, cat no. 66905-1-Ig), anti-vimentin RAb (1:2500; Proteintech, cat no. 10366-1-AP), anti-Zeb1 RAb (1:250; Proteintech, cat no. 21544-1-AP), anti-PRKACB RAb (1:200; Proteintech, cat no. 12232-1-AP) and anti-Ki67 MAb (1:50; Santa Cruz, sc-23900) overnight at 4°C. The slides were then incubated with a biotinylated anti-mouse secondary antibody (OriGene cat no. PV-9002) and biotinylated anti-rabbit antibody (OriGene, cat no. PV-9001) for 20 min at 37°C. The immunohistochemical analyses were performed simultaneously by two independent researchers.

### 2.5 Western blot analysis

Cell monolayers were lysed in RIPA buffer to obtain total cell protein extracts. Total protein was quantified using a BCA proteometric assay. Proteins were separated by 10% sodium dodecyl sulfate-polyacrylamide gel electrophoresis (SDS-PAGE) and transferred to a PVDF membrane. The membrane was incubated with the primary antibody at 4°C overnight. Then, the secondary antibody was added at room temperature for 45 min and the proteins were visualized using the FluorChenE Gel Imaging System (Protein Simple). For Western blot analysis, the primary antibodies anti-GLI1 MAb (1:2000; Proteintech, cat no. 66905-1-Ig), anti-PRKACB RAb (1:1000; Proteintech, cat no. 12232-1-AP), anti-Zeb1 (1:1000, Proteintech, cat no. 21544-1-AP), anti-vimentin (1:5000; Proteintech, cat no. 10366-1-AP), anti-GAPDH (1:5000; Proteintech, cat no. 10494-1-AP), anti-CD44 RAb (1:3000; Proteintech, cat no. 15675-1-AP) and anti-Sox2 RAb (1:2000; Cell Signaling, 23064S) were used. GAPDH was used as an internal standard.

### 2.6 Chromatin immunoprecipitation

Chromatin immunoprecipitation (ChIP) assays were performed using a ChIP Assay Kit (CST, USA). The manufacturer’s instructions were followed. Briefly, cells were cultured to approximately 1 × 10^7^ cells, cross-linked the proteins and DNA for 10 min at room temperature with 1% formaldehyde. The cells were harvested, washed twice with cold PBS, and lysed in SDS lysis buffer (50 mM Tris-HCl pH 8.0, 10 mM EDTA, 1% SDS) containing protease inhibitors. The lysates were sonicated using a sonicator to shear the DNA into fragments of 200–500 bp. The sonicated lysates were centrifuged at 14,000 rpm for 10 min at 4°C, and the supernatants were collected. The supernatants were pre-cleared by incubating with protein A/G agarose beads (CST) for 1 h at 4°C. After pre-clearing, the supernatants were divided into two aliquots and incubated with 2 µg of GLI (PTG) or IgG antibody per reaction overnight at 4°C with gentle rotation. Protein A/G agarose beads were added to the reactions and incubated for 2 h at 4°C to capture the antibody-DNA complexes. DNA was eluted from the beads by incubating with elution buffer (1% SDS, 0.1 M NaHCO3) for 15 min at room temperature twice. Real-time fluorescence quantitative PCR was performed to amplify the precipitated DNA using primers specific for the PRKACB promoter. The primer sequences were as follows: forward 5′-TTC​AGA​TCT​ACA​CAG​AAG​TTG​C-3′ and reverse 5′-CTC​GCA​CAT​TGA​GGT​ATT​CG-3’. Non-immunoprecipitated chromatin fragments were used as the input controls, and the relative enrichment of the target DNA was calculated using the comparative Ct method.

### 2.7 Wound-healing assay

Cell migration was evaluated using a wound-healing assay. Briefly, 2 × 10^5^ AGS or HGC cells per well were seeded in six-well plates. After the cells reached approximately 90% confluence, the cell monolayers were wounded vertically with a 20 µL or 200 µL sterile pipette tip. The wells were washed with phosphate buffered saline (PBS) and fresh medium was added. The plates were placed in a 37°C incubator with 5% CO_2_, and the migrated cells were observed and photographed at 0 h and 24 h after scratching using an Olympus CKX41 microscope. The width of the wound was measured using image analysis software (ImageJ), and the migration rate was calculated as the ratio of the decrease in wound width to the initial wound width.

### 2.8 Cell migration and invasion assay

The cell migration assay was conducted using 24-well plates with 8-μm pore Transwell chambers (Corning, United States). A total of 1 × 10^5^ AGS or HGC cells were placed in the upper chamber in serum-free medium. The lower chamber was filled with culture medium containing 20% FBS. After 24 h, the non-migrated cells were removed with clean swabs and the cells on the bottom surface were stained with 0.1% crystal violet dye. For the cell invasion assay, AGS or HGC cells were seeded at a density of 1 × 105 cells/well in the upper chamber filled with Matrigel (Corning, United States). After 24 h, non-invading cells were removed with swabs and the cells on the underside were stained with 0.1% crystal violet dye. The migration and invasion abilities of AGS and HGC cells were quantified by inverted contrast microscopy.

### 2.9 Cell sphere-forming assay

The cells were digested with trypsin and resuspended for cell counting, and 5 × 10^3^ cells were spread in a low-adhesion 6-well plate with three replicate wells. The plate was placed in a 37°C incubator with 5% CO_2_ and cultured for 7 days. During the culture process, the medium was not changed. After 7 days of culture, photographs were taken under a microscope, and five fields were randomly selected to count the number of sphere-forming cells using image analysis software (ImageJ). The sphere formation efficiency was calculated as the ratio of the number of sphere-forming cells to the total number of seeded cells.

### 2.10 EdU incorporation assay

The assay was carried out according to the instructions of the Cell‐Light EdU Apollo 567 *In Vitro* kit (Ribobio Ltd, China). In 96‐well plates, 5 × 10^3^ cells were added to each well and cultured overnight. 100 μL culture medium (containing 0.1% EdU) was added to each well for 2 h before fixation in 4% paraformaldehyde. After permeabilization with 0.5% Triton X‐100, 100 μL 1×Apollo‐staining reaction liquid was added to cells at 37°C for 30 min, then the cells were counterstained with 4′,6‐diamidino‐2‐phenylindole (DAPI) and imaged using a fluorescence microscope (Olympus, Tokyo, Japan). The images were analyzed using image analysis software (ImageJ), and the percentage of proliferating cells (EdU-positive cells) was calculated as the ratio of the number of EdU-positive cells to the total number of DAPI-stained cells.

### 2.11 Cell clone formation test

In a 24‐well plate, 1000 cells were added to each well. The cells were cultured in the complete culture medium described above for 10 days. During the culture process, the medium was changed every 3 days. After 10 days of incubation, 500 μL methanol was added to each well for 15 min. After washing twice with PBS buffer, each well was stained with 0.1% crystal violet dye for 30 min.The colonies were counted under a light microscope, and the clone formation efficiency was calculated as the ratio of the number of colonies to the number of seeded cells.

### 2.12 Circular dichroism (CD)

The purified protein was subjected to SDS-PAGE, and the stained and decolorized gels were used to determine the purity of the target protein. The gel was stained with Coomassie Brilliant Blue R-250 and then decolorized using a destaining solution (a mixture of methanol, acetic acid, and water). The purified protein can be used for CD spectroscopy when the content of target protein is 95% or more. The spectra were recorded in the far-ultraviolet, 190–240 nm, at room temperature using a circular dichroic chromatograph. The concentration of the sample was 100 μg/mL and the optical diameter of the sample cup was 0.2 cm. The resolution was 0.5 nm, bandwidth of 0.5 nm, sensitivity of 50 mdeg, and speed of 0.8 nm/min. Secondary structures of the peptides were calculated using Yang’s algorithm (Yang. Jsr). The algorithm analyzes the CD spectra data and estimates the proportions of different secondary structures.

### 2.13 Dual-luciferase assay

Human gastric cancer cells were plated in 6-well plates at a density of 2 × 10^5^ cells per well and cultured in the complete culture medium described above for 24 h. Then, the cells in each well were transfected with 1 μM plasmid (including the wild-type or mutant GLI1 promoter reporter plasmid and the control Renilla luciferase plasmid). Briefly, the plasmid DNA was diluted in Opti - MEM medium (Gibco), and Lipofectamine 2000 was added at a ratio of 1:2 (μg of DNA to μL of Lipofectamine 2000). The mixture was incubated at room temperature for 20 min to form transfection complexes. Subsequently, the transfection complexes were added to the cells dropwise, and the cells were incubated in a 37°C incubator with 5% CO_2_ for 6 h. After that, the medium was replaced with fresh complete culture medium. After transfection, the cells were harvested 24 h after drug treatment and manipulated using a Dual-Glo kit (Promega, 0000551855). Assays were performed using the Dual-Luciferase Assay Luciferase Assay System (Promega) according to the manufacturer’s protocol. The firefly/renal luciferase ratio was calculated as the normalized reporter gene activity, with the control value set at 1.0. Each experiment was repeated at least three times to ensure the reliability of the data.

### 2.14 Animal experiments

The animal assays were approved by the Institutional Animal Care and Use Committee of the Central Hospital affiliated with Shandong First Medical University. BALB/c nude mice (4 weeks old, male, 18–20 g) were purchased and acclimatized to the experimental conditions for 1 week. The mice were randomly divided into control and experimental groups. The experimental groups were alpha-estradiol (n = 6), (R)-(−)-ibuprofen (n = 6), GANT61 (n = 6), cisplatin (n = 6), and cisplatin in combination with (R)-(−)-ibuprofen (n = 6). A total of 2 × 106 HGC cells suspended in 100 μL of medium were injected intravenously (IV), or 1 × 10^7^ HGC cells suspended in 50 μL of medium mixed with 50 μL Matrigel Basement Membrane Matrix High Concentration (Corning 354248) were injected subcutaneously (SC) into the BALB/c nude mice. The drug was injected intraperitoneally daily for 1 week. On days 5, 7, 9, 11, 13, and 15, the tumor size in each mouse was measured. The mice were subsequently sacrificed, and the tumor tissue was collected for analysis.

### 2.15 Statistical analysis

All data were analyzed and graphed using GraphPad Prism 7.0. Data were expressed as (mean ± SD). Group t-tests were used for two independent samples. The relationship between GLI1 and PRKACB expression and pathological parameters was examined using the Fisher’s exact test. Survival analysis was performed by Kaplan–Meier curves. Statistical significance was set at P < 0.05.

## 3 Results

### 3.1 GLI1 expression in the gastric cancer dataset and patients

**FIGURE 1:**
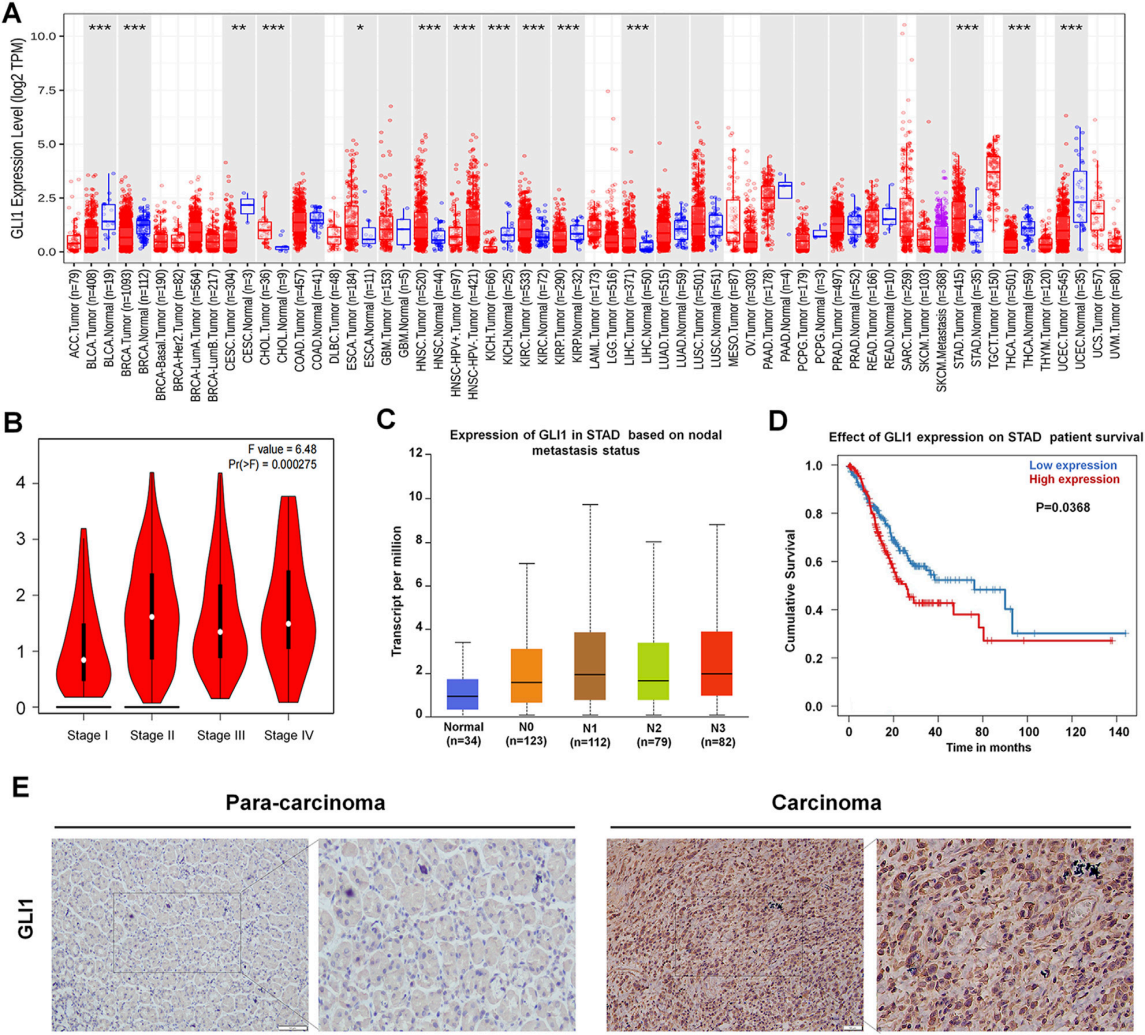
GLI1 expression in gastric cancer. **(A)** GLI1 expression in various cancers, from the TIMER database. **(B)** GLI1 expression is correlated with clinical stages. **(C)** GLI1 expression is correlated with lymph node metastasis status. **(D)** GLI1 expression is correlated with poor prognosis. **(E)** GLI1 expression in patients with gastric cancer.

A TIMER database was used to analysis the expression of GLI1 in various cancer. GLI1 expression significantly increased in gastric cancer, kidney renal clear cell carcinoma and hepatocellular carcinoma and we selected GC for our study (Figure 1A). For gastric cancer in the TCGA database, high GLI1 expression was significantly associated with clinical stage and lymph node status (Figures 1B, C). A survival analysis of patients with high GLI1 expression, using the UALCAN online database showed that GLI1 expression was associated with poor prognosis in patients with GC (Figure 1D). In addition, GLI1 protein level in gastric cancer tissues was significantly higher than that in tumor-adjacent tissues, which is consistent with the results of previous studies (Figure 1E).

### 3.2 Alpha-estradiol and (R)-(−)-ibuprofen as G-quadruplex stabilizers of the GLI1 promoter

**FIGURE 2:**
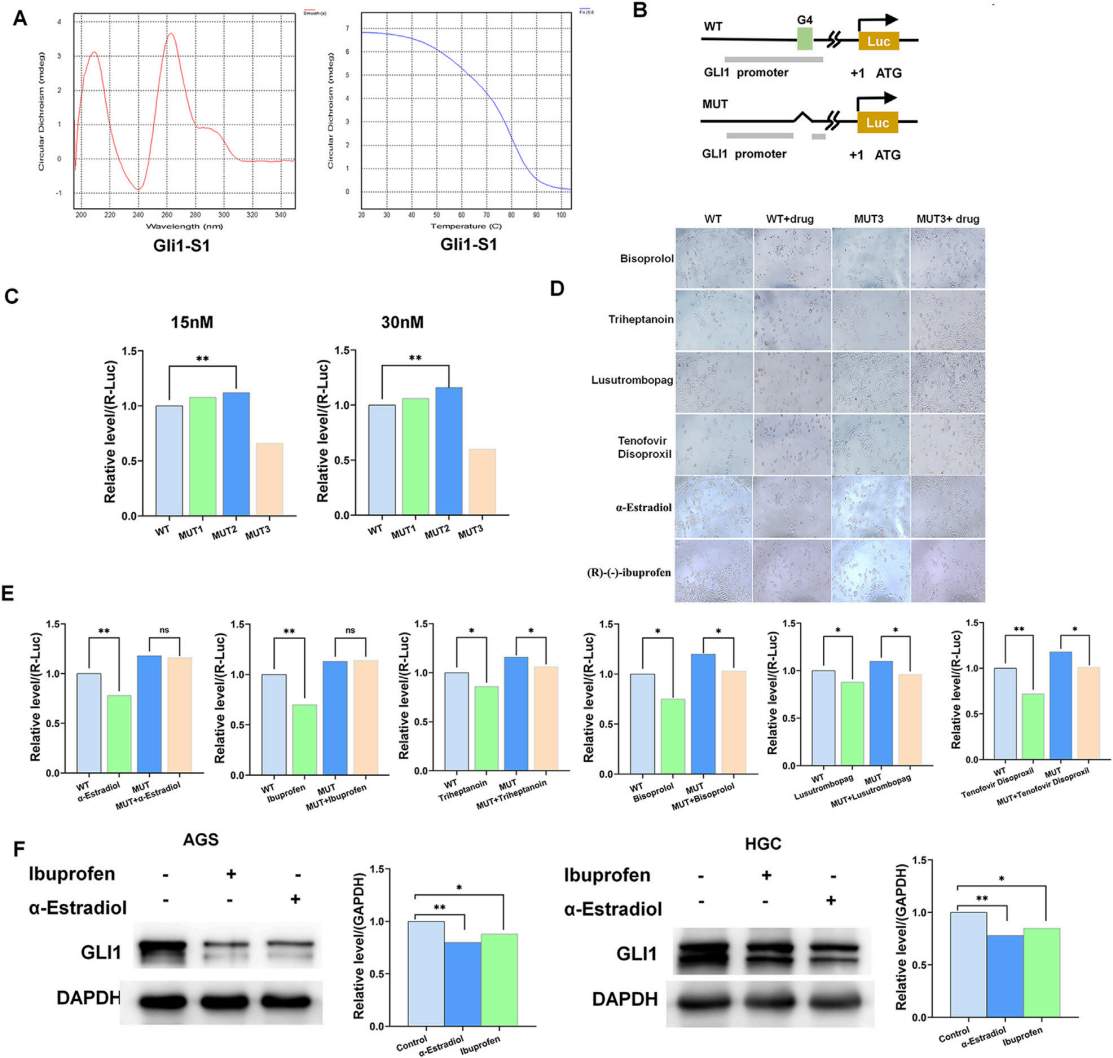
α-Estradiol and ibuprofen as the G-quadruplex stabilizers of the GLI1 promoter mediate cell migration and invasion in gastric cancer. **(A)** Circular dichroism spectrum of GLI1 G-quadruplex. **(B, C)** The activity of the G-quadruplex in HGC cells determined by luciferase reporter assays. **(D, E)** Alpha-estradiol and ibuprofen stabilize the G-quadruplex, to inhibiting gastric cancer cell proliferation. **(F)** GLI1 expression is inhibited by alpha-estradiol and ibuprofen treatment.

Circular dichroism (CD) analysis revealed that the GLI1 promoter region can form G-quadruplex structures (Figure 2A). We mutated the region of the GLI1 promoter that readily formed G-quadruplexes and screened the best mutated fragments using a dual-luciferase reporter assay (Figures 2B, C). Using the FDA drug library (Selleck, Cherry pick libarary, Z590771), we initially screened six drugs that only targeted wild-type gastric cancer cells but had no effect on mutated-type gastric cancer cells (Figure 2D). To further demonstrate the effects of these drugs, we validated them by using a dual-luciferase reporter assay. The results showed that alpha-estradiol (α-Estradiol) and (R)-(−)-ibuprofen significantly reduced the expression of GLI1 transcripts (Figure 2E). In addition, Western blotting results showed that the protein level of GLI1 was significantly reduced with the addition of alpha-estradiol and (R)-(−)-ibuprofen compared to that in the control group (Figure 2F). These results suggest that α-estradiol and (R)-(−)-ibuprofen can act as G-quadruplexes stabilizers on the GLI1 promoter to influence the state of gastric cancer cells.

### 3.3 Alpha-estradiol and (R)-(−)-ibuprofen inhibit the proliferation, migration and invasion of gastric cancer cells *in vitro*

**FIGURE 3:**
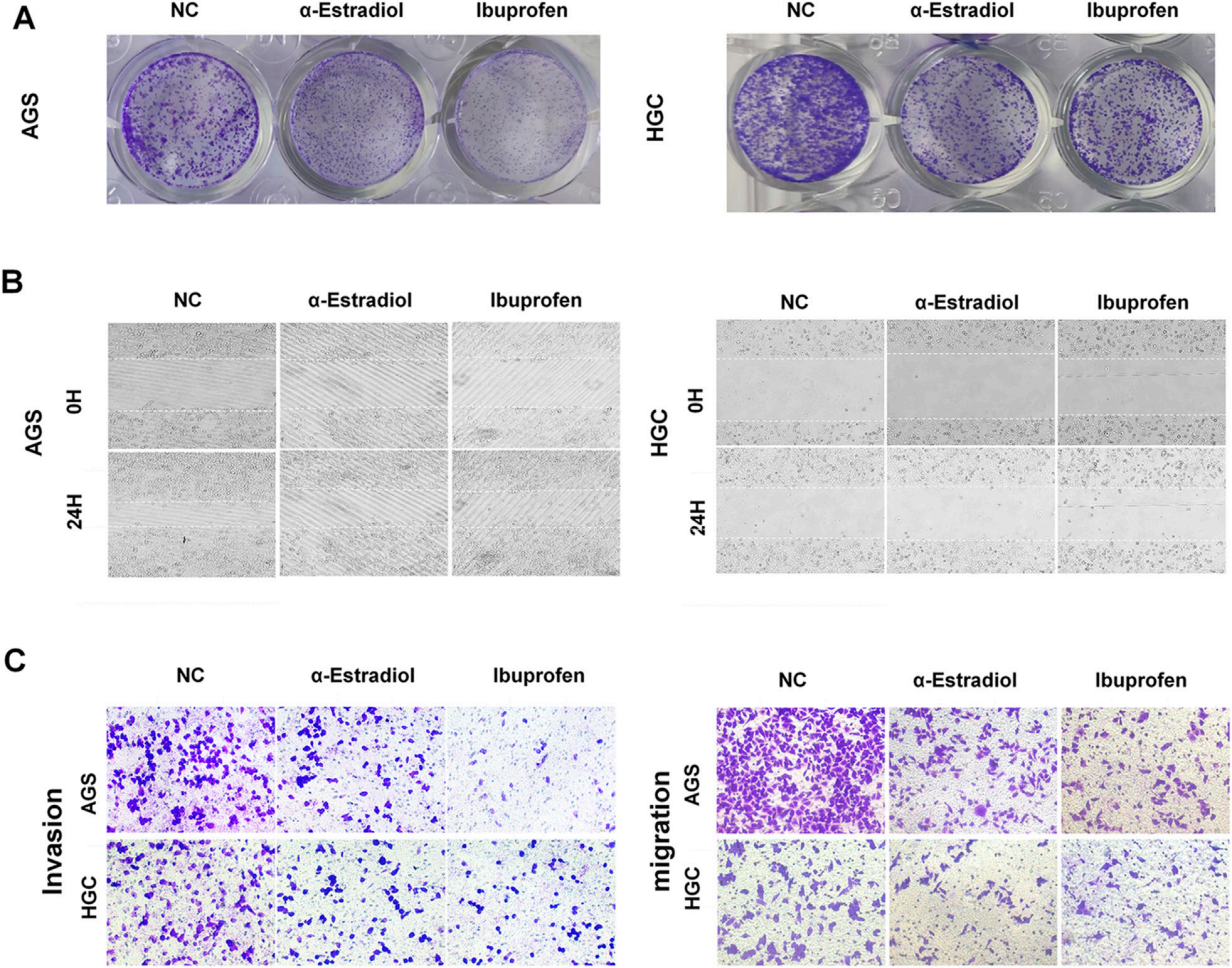
Alpha-estradiol and ibuprofen mediated gastric cancer cell migration and invasion. **(A)** Alpha-estradiol and ibuprofen inhibit cell proliferation in the colony-formation assay. **(B)** Alpha-estradiol and ibuprofen inhibit cell migration in the wound healing assay. **(C)** Alpha-estradiol and ibuprofen inhibit cell invasion in the transwell assay.

To validate the effects of alpha-estradiol and (R)-(−)-ibuprofen, a series of *in vitro* experiments were conducted. The results showed that the cell proliferation rate was significantly reduced in the groups treated with alpha-estradiol or (R)-(−)-ibuprofen (Figure 3A). Wound healing was significantly slower than that in the group without the addition of estradiol (Figure 3B). Similarly, the migration and invasion of AGS and HGC cells were significantly reduced by the addition of alpha-estradiol and (R)-(−)-ibuprofen compared to those in the control group (Figure 3C). These results show that alpha-estradiol and (R)-(−)-ibuprofen inhibit the migration and invasion of gastric cancer cells by regulating the stability of G-quadruplexes in the GLI1 promoter.

### 3.4 Alpha-estradiol and (R)-(−)-ibuprofen inhibit the growth of gastric cancer *in vivo*

**FIGURE 4:**
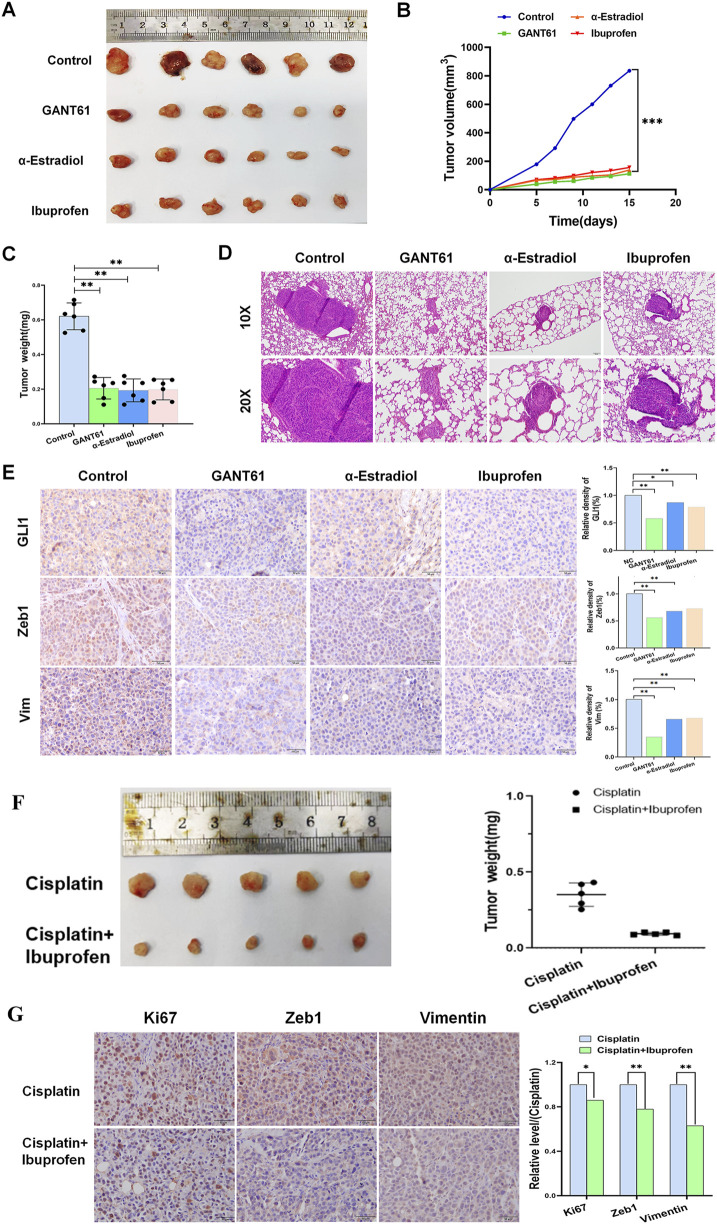
Alpha-estradiol and ibuprofen suppress gastric cancer cell growth *in vivo*. **(A)** Representative tumor size of isolated tumors from mice injected with HGC cells. **(B)** HGC cell-derived xenograft tumor volume measurements. **(C)** HGC cell-derived xenograft tumor weight from mice. **(D)** Representative hematoxylin and eosin staining of lung from HGC cell-derived xenograft mice with GANT61, alpha-estradiol and ibuprofen treatment. **(E)** GLI1, Zeb1, and Vimentin expression in GANT61, alpha-estradiol and ibuprofen treatment groups. **(F)** Ibuprofen in combination with Cisplatin decreased tumor size and weight compared with Cisplatin alone. **(G)** The expression of Ki67, Zeb1, Vimentin decreased in combination treatment. *P < 0.05, **P < 0.01.

To further validate the regulatory functions of alpha-estradiol and (R)-(−)-ibuprofen in gastric cancer growth, we implanted human HGC cells into BALB/c nude mice. *In vivo*, the results showed that alpha-estradiol and (R)-(−)-ibuprofen significantly inhibited tumor growth. This was consistent with the results obtained using GANT61, a laboratory-recognized GLI1 inhibitor (Figure 4A). In addition, there was a corresponding improvement in tumor volume and weight after treatment with alpha-estradiol and (R)-(−)-ibuprofen (Figures 4B, C). The effects of alpha-estradiol and (R)-(−)-ibuprofen on invasion and metastasis of gastric cancer cells were investigated by tail vein injection experiments. The results showed that alpha-estradiol and (R)-(−)-ibuprofen inhibited the lung metastasis of gastric cancer cells (Figure 4D). In addition, immunohistochemical analysis of tumor tissues showed that alpha-estradiol and (R)-(−)-ibuprofen inhibited the expression of GLI1, Zeb1 and Vimentin (Figure 4E). These data confirmed that GLI1-mediated tumor growth and metastasis in gastric cancer can be inhibited by alpha-estradiol and (R)-(−)-ibuprofen.

Given the clinical roles of these two drugs, we speculated that they could be used as adjuvants to enhance the efficacy of chemotherapy drugs. The results showed that cisplatin treatment in combination with (R)-(−)-ibuprofen was more effective than cisplatin treatment alone (Figure 4F). In addition, immunohistochemistry results showed that the combination therapy further reduced the expression of Zeb1 and Ki67, which was consistent with previous results (Figure 4G).

### 3.5 GLI1 is highly correlated with PRKACB in gastric cancer in the dataset and in gastric xenograft tissues treated with alpha-estradiol and (R)-(−)-ibuprofen

**FIGURE 5:**
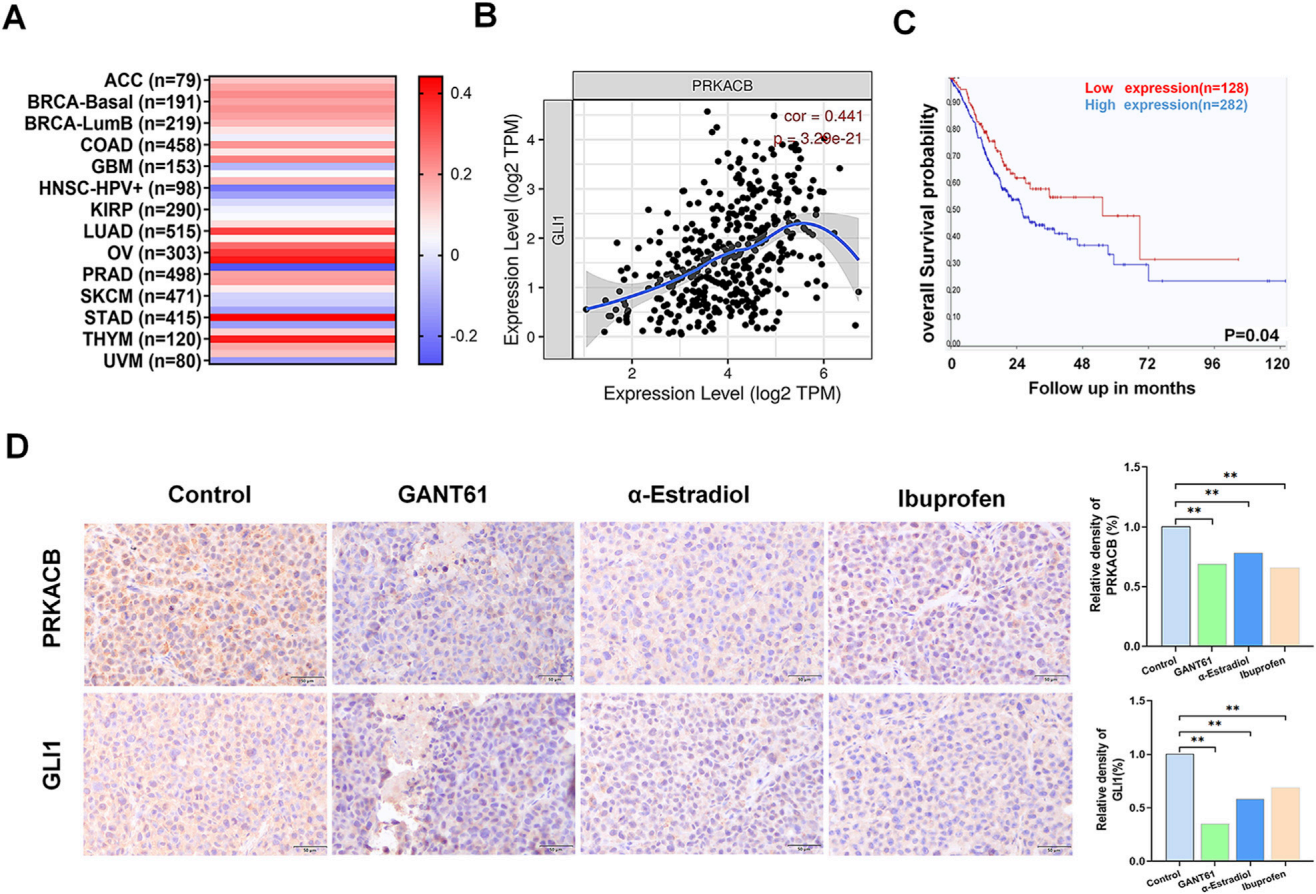
GLI1 and PRKACB expressions are correlated in gastric cancer. **(A)** Correlation between GLI1 and PRKACB expression in various cancers; **(B)** The association of GLI1 expression with PRKACB. **(C)** PRKACB expression correlates with poor prognosis. **(D)** GLI1 and PRKACB expression in GANT61, alpha-estradiol and ibuprofen treatment groups in gastric cancer and tumor-adjacent tissues. Scale bars 100 μm *P < 0.05.

It was reported that PRKACB expression was elevated in gastric cancer tissues and was associated with tumor development. To verify whether there was an association between GLI1 and PRKACB, we analyzed the data using the TIMER database and found that the correlation coefficient between PRKACB and GLI1 expression in gastric cancer was the highest amongst the analyzed tumors (Figures 5A, B). Patients with high PRKACB expression showed poorer survival rates (Figure 5C) and significantly correlated with age, Lauren stage, PNI, TNM stage, pathological stage, and Borrmann stage (Table 1). In addition, the expression levels of GLI1 and PRKACB were reduced after treatment with alpha-estradiol and (R)-(−)-ibuprofen in gastric xenograft tissues (Figure 5D). These results suggested that GLI1 may be related to PRKACB expression in gastric cancer.

**TABLE 1:** PRKACB expression correlated with clinical parameters in lung adenocarcinoma specimens.

Clinicopathological parameters		Cases	High	Low	P
Sex	Male	199	92	107	0.067
	Female	101	58	43	
Age	<60	106	65	41	0.004*
	≥60	194	85	109	
EBV	-	257	128	129	0.354
	+	18	11	7	
LAUREN	Intestinal	150	54	96	0.001*
	Diffuse	142	91	51	
	Mixed	8	5	3	
PNI	-	159	67	92	0.011*
	+	88	52	36	
Venous invasion	-	129	65	64	0.277
	+	44	18	26	
TNM (T)	T1, T2	188	77	111	0.001*
	T3, T4	112	73	39	
TNM(N)	N0, N1	169	81	88	0.415
	N2, N3	131	69	62	
TNM(M)	M0	273	131	142	0.027*
	M1	27	19	8	
pStage	I II	126	53	73	0.015*
	III IV	172	97	75	
Borrmann	I	16	6	10	0.001*
	II	102	40	62	
	III	140	70	70	
	IV	38	32	6	
Revised location	antrum	150	69	81	0.468
	body	107	57	50	
	cardia	30	16	14	
Recurrence	-	151	70	81	0.144
	+	125	69	56	

### 3.6 GLI1 mediates PRKACB expression in gastric cancer *in vitro*

**FIGURE 6:**
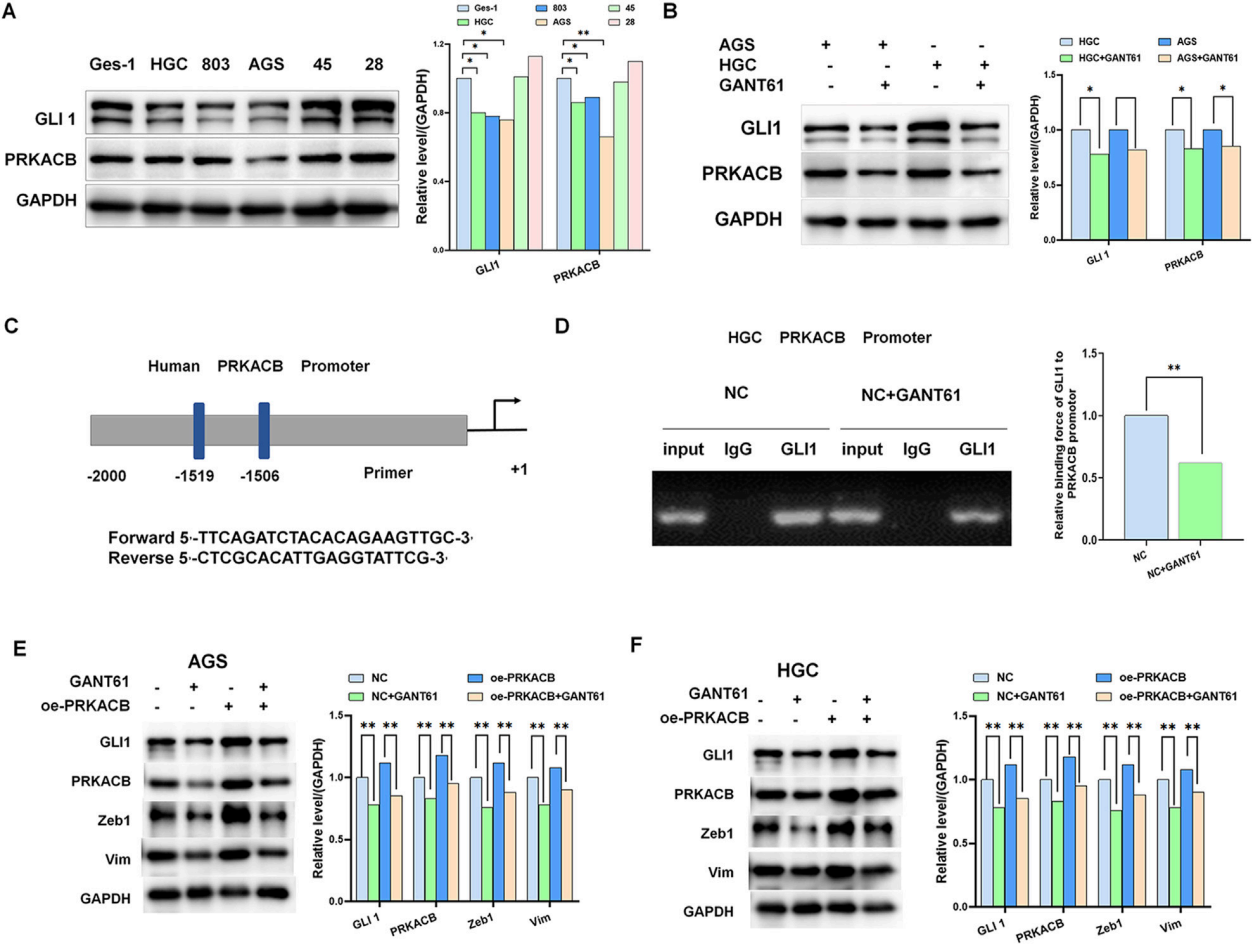
GLI1 mediates PRKACB expression in gastric cancer *in vitro*. **(A)** Expression of GLI1 and PRKACB in gastric cancer cell lines. **(B)** GANT61 inhibits PRKACB expression in HGC and AGS gastric cancer cell lines. **(C, D)** Chromatin immunoprecipitation assay showing GLI1 binding to the PRKACB promoter in gastric cancer. **(E, F)** GANT61 downregulated the expression of PRKACB and Zeb1 in gastric cancer. *P < 0.05, **P < 0.01.

To verify the relationship between GLI1 and PRKACB, we selected AGS and HGC cell lines with low PRKACB expression (Figure 6A). After adding GANT61, we found that PRKACB expression decreased, along with a reduction in GLI1 expression (Figure 6B). GLI1 acts as a transcription factor that regulates the expression of several molecules. We speculate that GLI1 directly binds to the promoter region of PRKACB and regulates its expression. Transcriptional element analysis (http://jaspar.genereg.net) revealed that the transcription factor GLI1 directly bound to the PRKACB promoter at −418 to −408 (Figure 6C). Next, we conducted ChIP. The results showed that GLI1 could bind to the promoter of PRKACB, and that this binding was reduced upon the addition of GANT61 (Figure 6D). The addition of GANT61 to the control and PRKACB overexpression groups significantly suppressed epithelial mesenchymal transition markers compared with the control and PRKACB overexpression groups (Figures 6E, F). Taken together, these findings suggest that GLI1 mediates PRKACB expression in gastric cells *in vitro*.

### 3.7 GLI1/PRKACB signaling promotes the proliferation, migration, invasion and stem cell-like properties of gastric cancer cells

**FIGURE 7:**
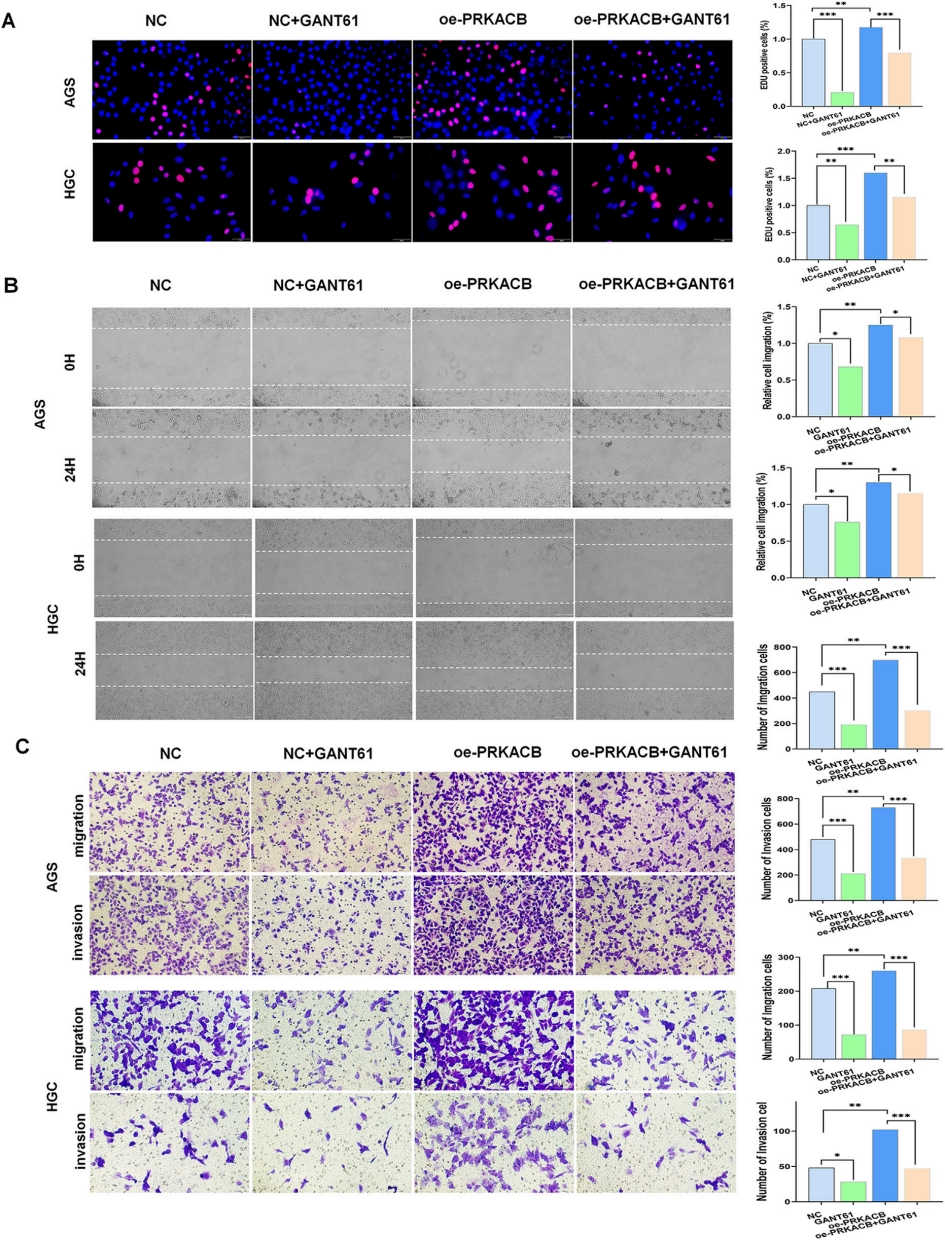
The expression of GLI1 and PRKACB synergistically affects cell proliferation, migration, invasion and stem cell-like properties in HGC and AGS cells. **(A)** EdU incorporation assay shows that silencing GLI1 and/or PRKACB reduces cell proliferation. Red denotes positive spots, while blue (DAPI) indicates nuclei (n = 3). Scale bar, 100 µm. **(B)** Wound healing assay shows that GLI1 and/or PRKACB downregulation inhibit the migration of HGC and AGS cells. Scale bar, 100 µm (n = 3). *P < 0.05, **P < 0.01. **(C)** Transwell assays show that GLI1 and/or PRKACB downregulation inhibit the invasion of HGC and AGS cells. Scale bar, 100 µm (n = 3). *P < 0.05, **P < 0.01.

Both GLI1and PRKACB have been reported to significantly promote the proliferation, migration, and invasion of cancer cells. The EdU assay showed that the proliferation of the GANT61 and overexpressing (oe)-PRKACB + GANT61 groups was significantly lower than that of the control and oe-PRKACB groups (Figure 7A). Wound healing in the GANT61 and oe-PRKACB + GANT61 groups was significantly slower than in the control and oe-PRKACB groups (Figure 7B). The GANT61 and oe-PRKACB + GANT61 groups showed reduced migration and invasion of AGS and HGC cells compared to the control and oe-PRKACB groups (Figure 7C).

**FIGURE 8:**
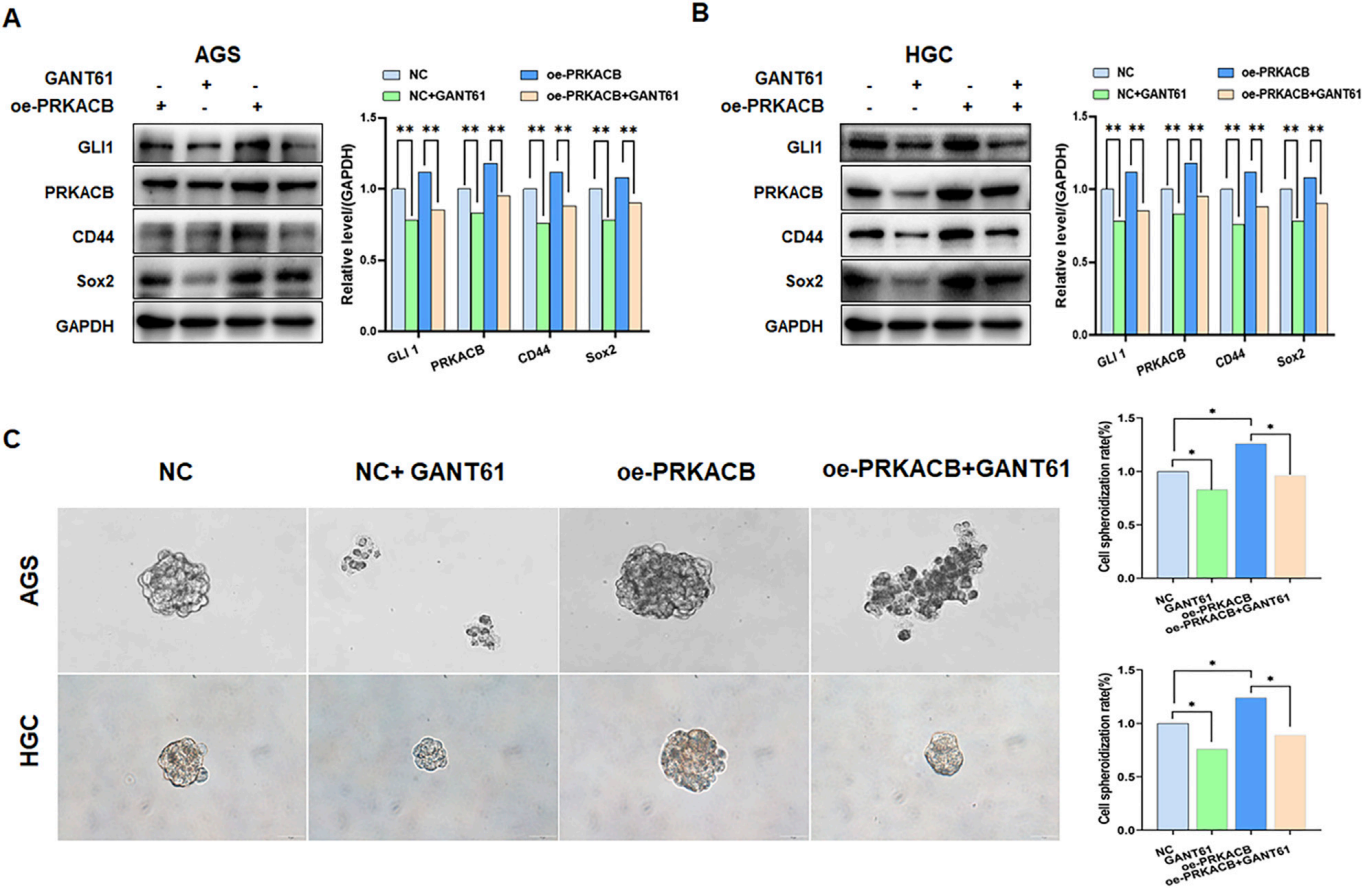
Expression of GLI1and PRKACB affects stem cell-like properties in HGC and AGS cells. **(A, B)** Inhibiting GLI1 or/and overexpressing PRKACB decreases CD44 in AGS and HGC cells. **P < 0.01. **(C)** Representative spheroid images formed by single cells with inhibition of GLI1 expression and/or overexpressing PRKACB.

GLI1 and PRKACB are highly correlated with cell stemness, so we further explored the correlation with cancer cell stemness. SOX2 and CD44 are markers for cancer stem cells. The levels of CD44 and SOX2 decreased after treatment with GANT61 in AGS and HGC cell lines (Figures 8A, B). The sphere formation assay demonstrated that sphere formation efficiency was significantly attenuated in the GANT61 and oe-PRKACB + GANT61 groups compared to that in the control and oe-PRKACB groups in AGS and HGC cells (Figure 8C). These results suggested that GLI1 and PRKACB are involved in gastric cancer cell stemness.

### 3.8 GLI1 and PRKACB expression in gastric cancer in tissue microarray

**FIGURE 9:**
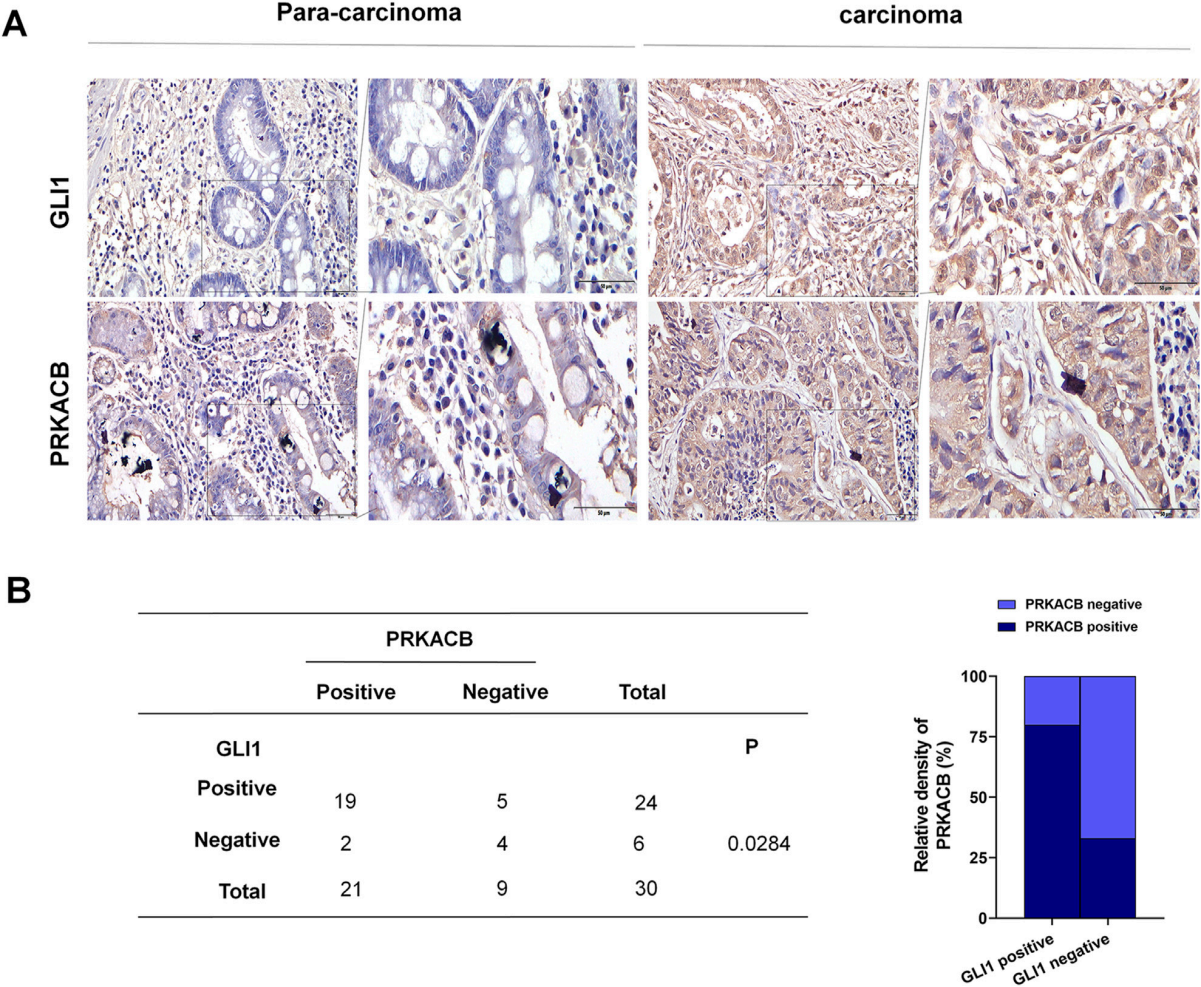
PRKACB and GLI1 expression in gastric cancer tissue microarray. **(A)** GLI1 and PRKACB expression in gastric cancer carcinoma and tumor-adjacent tissues. **(B)** Correlation between GLI1 and PRKACB expression in gastric cancer tissues. Scale bars are 100 µm.

GLI1 and PRKACB expression was examined using immunohistochemistry in tissue microarrays containing 30 gastric cancer and 30 tumor-adjacent tissue samples. GLI1 protein levels were significantly higher in gastric cancer tissues than in tumor-adjacent tissue samples, consistent with PRKACB protein levels in gastric cancer tissues (Figure 9A). Spearman’s correlation analysis revealed a correlation between GLI1 and PRKACB expression levels (Figure 9B) (P < 0.05). The data from the tissue microarrays suggest that GLI1 is strongly associated with PRKACB in patient samples.

## 4 Discussion

Gastric cancer is a serious threat to human health. According to the 2021 statistics compiled by the American Cancer Society, incidence and mortality rates remain high (Siegel et al., 2021). The treatment of gastric cancer is multidisciplinary and comprehensive, including endoscopic treatment, surgery, radiotherapy, and systemic chemotherapy. However, the efficiency and side effects of these methods limit their clinical application, and targeted therapy, as a widely recognized direction of research on the disease, has been gradually applied to clinical research (Alsina et al., 2023). Drugs that specifically target nucleic acid structures through binding, intercalation, or covalent modification have been developed to treat various diseases (Tateishi-Karimata and Sugimoto, 2020). The impact of G4 structures on DNA and RNA functions presents an enticing opportunity to modulate these folded structures and exert control over their diverse functions. The distinctive structural features of G4 have facilitated the development of diverse structure-selective small molecule ligands (Carvalho et al., 2020; Berner et al., 2024). In this project, we combined previous studies with the current research status and used the GLI1 molecule in the Hh pathway as a regulatory molecule to develop inhibitors for G-quadruplexes, providing a new option for the treatment of gastric cancer. Furthermore, we confirmed that G-quadruplex regulators of the GLI1 promoter inhibited gastric cancer progression by downregulating PRKACB, an effector molecule downstream of GLI1.

Aberrant activation of Hh signaling is observed in many tumors and accounts for approximately 25% of human cancer deaths (Lum and Beachy, 2004). The Hh pathway is active in stem cells and largely inactive in adult organisms. Aberrant activation of this pathway in adult cells affects the downstream cellular processes and leads to the development of multiple cancers, including gastric cancer, tissue infiltration, and drug resistance (Karhadkar et al., 2004; Zhu et al., 2019; Huang et al., 2006). GLI is a transcription factor belonging to the zinc finger protein family and a major effector of Hh signaling (Doheny et al., 2020). In vertebrates, there are three members of the GLI gene family: GLI1, GLI2 and GLI3 (Niewiadomski et al., 2019). GLI1 can act as a transcriptional activator (Hynes et al., 1997) and Hh ligands can induce GLI1 expression, which provides a positive feedback loop for Hh signaling (Regl et al., 2002). In human gliomas the Hh/GLI1 pathway plays an important role in the self-renewal and tumorigenicity of cancer stem cell-like cells (Clement et al., 2007). Our study showed that GLI1 was overexpressed in gastric cancer tissue samples and that its expression was correlated with adverse clinicopathological parameters, suggesting a role for GLI1 in gastric carcinogenesis, progression, and metastasis. By far, no molecular inhibitor of GLI1 has been approved for the treatment of gastric cancer. Attempting to block or silence the expression of GLI1 will significantly help suppress gastric cancer progression.

In recent years, the exploration of nucleic acid secondary structures has drawn increasing attention. A G-quadruplex is a nucleic acid secondary structure usually found in telomeres, or the promoter region of a gene, and is formed by the folding of a sequence rich in guanine bases (Zhang et al., 2022) (Kosiol et al., 2021). The formation of G-quadruplexes is closely related to biological processes such as gene expression regulation, genome replication, and telomere elongation, and influences cellular senescence and apoptosis, which are involved in the onset and progression of cancer and other diseases (Tan et al., 2018; Hirashima and Seimiya, 2015; Murat and Balasubramanian, 2014; Huppert and Balasubramanian, 2005). G-quadruplexes located in the promoter and 5′-untranslated regions play a regulatory role in gene expression, and the formation of G-quadruplexes tends to repress the expression of oncogenes such as MYC, FGR, hTERT, and BCL2 (Hänsel-Hertsch et al., 2017; Hänsel-Hertsch et al., 2018; Lin et al., 2024). Therefore, these G-quadruplex structures may serve as potential targets for anticancer drugs. The guanine-rich sequence in the promoter region of GLI1 forms a G-quadruplex structure, which is a good target for inhibitor development. In this study, through meticulous molecular ligand screening, two inhibitors, namely, alpha-estradiol and (R)-(−)-ibuprofen, were identified. Our investigations revealed that these inhibitors could effectively modulate GLI1 expression and consequently intervene in the progression of gastric cancer, opening up new avenues for future therapeutic strategies.

Our results have showed that G-quadruplex regulators on the GLI1 promoter inhibited gastric cancer progression by downregulating PRKACB, an effector molecule downstream of GLI1. PRKACB is located at chromosome 1p31.1, has a total gene length of 160 kb, and is a member of the serine/threonine protein kinase family (Zhu et al., 2005). As an important molecule in the cellular cascade signaling pathway, the activated catalytic subunit can transmit extracellular stimulatory signals and participate in the regulation of cell proliferation, differentiation and apoptosis (Skalhegg and Tasken, 2000). It is a key link in the regulation of the cell cycle and is closely related to cell growth, differentiation, gene expression, tumor growth, metastasis, and other physiological and pathological processes (Yao et al., 2020; Higuchi et al., 2003; Kvissel et al., 2007). Our study showed that PRKACB is highly expressed in gastric cancer and acts as a downstream regulator of GLI1 to promote gastric cancer progression.

There are some limitations of the present study that could be addressed in our following study. G-quadruplex has potential as a drug target, but due to its wide distribution and diversity in the genome, developing GLI1 G-quadruplex targeted drugs with high specificity and minimal side effects still faces challenges. The clinical value should be further confirmed in a larger cohort including preclinical intervention studies. To further explore the apoptosis and the changes in the phases of the cell cycle via the GLI1/PRKACB pathway following treatment with these inhibitors would provide a more comprehensive understanding of the mechanisms.

## 5 Conclusion

**FIGURE 10:**
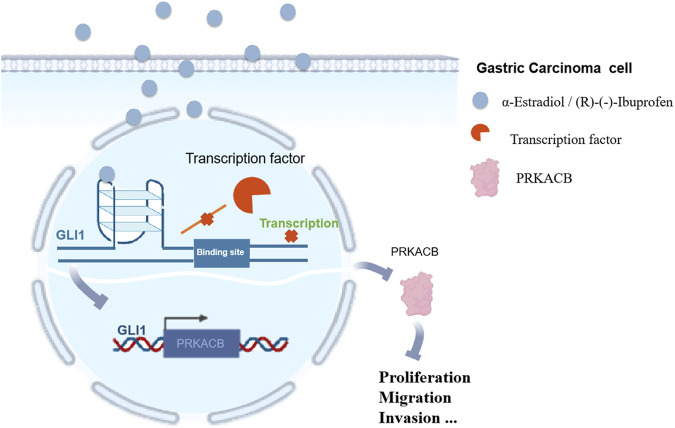
GLI1 G-quadruplex regulators inhibit gastric cancer progression via down-regulating PRKACB.

In this study, a G-quadruplex was used as a target for inhibitor exploration, and a GLI1 molecule inhibitor was used as an entry point to verify the relationship between its aberrant activation and gastric cancer progression. In addition, G-quadruplex regulators of the GLI1 promoter inhibited gastric cancer cell proliferation, migration, and invasion by downregulating PRKACB, which provides more options for the clinical treatment of gastric cancer (Figure 10).

## Data Availability

The data supporting the findings of this study are available from the corresponding author upon reasonable request.
